# Liquid Biopsy and Multi-Omic Biomarkers in Breast Cancer: Innovations in Early Detection, Therapy Guidance, and Disease Monitoring

**DOI:** 10.3390/biomedicines13123073

**Published:** 2025-12-12

**Authors:** Daniel Simancas-Racines, Náthaly Mercedes Román-Galeano, Juan Pablo Vásquez, Dolores Jima Gavilanes, Rupalakshmi Vijayan, Claudia Reytor-González

**Affiliations:** 1 Facultad de Salud y Bienestar, Pontificia Universidad Católica del Ecuador, Quito 170143, Ecuador; 2 Facultad de Ciencias Médicas, de la Salud y la Vida, Universidad Internacional del Ecuador UIDE, Quito 170411, Ecuador; nathalyroman0001@gmail.com; 3 Independent Researcher, Quito 170102, Ecuador; juanpyfg@gmail.com; 4 Escuela de Medicina, Universidad Espíritu Santo, Samborondón 0901952, Ecuador; doloresjima78@hotmail.com; 5 St. Elizabeth Boardman Hospital, Boardman, OH 44512, USA; rvijayan@mercy.com; 6 Escuela de Medicina, Pontificia Universidad Católica del Ecuador, Santo Domingo 230203, Ecuador

**Keywords:** breast cancer, multi-omic biomarkers, liquid biopsy, precision medicine, healthcare

## Abstract

Liquid biopsy and multi-omic biomarker integration are transforming precision oncology in breast cancer, providing real-time, minimally invasive insights into tumor biology. By analyzing circulating tumor DNA, circulating tumor cells, exosomal non-coding RNAs, and proteomic or metabolomic profiles, clinicians can monitor clonal evolution, therapeutic response, and recurrence risk in real time. Recent advances in sequencing technologies, methylation profiling, and artificial intelligence–driven data integration have markedly improved diagnostic sensitivity and predictive accuracy. Multi-omic frameworks combining genomic, transcriptomic, and proteomic data enable early detection of resistance, molecular stratification, and identification of actionable targets, while machine learning models enhance outcome prediction and therapy optimization. Despite these advances, key challenges persist. Pre-analytical variability, lack of standardized protocols, and disparities in access continue to limit reproducibility and clinical adoption. High costs, incomplete regulatory validation, and the absence of definitive evidence for mortality reduction underscore the need for larger, prospective trials. Integrating multi-omic assays into clinical workflows will require robust bioinformatics pipelines, clinician-friendly reporting systems, and interdisciplinary collaboration among molecular scientists, data engineers, and oncologists. In the near future, liquid biopsy is expected to complement, not replace, traditional tissue analysis, serving as a cornerstone of adaptive cancer management. As sequencing becomes faster and more affordable, multi-omic and AI-driven analyses will allow earlier detection, more precise treatment adjustments, and continuous monitoring across the disease course. Ultimately, these innovations herald a shift toward real-time, data-driven oncology that personalizes breast cancer care and improves patient outcomes.

## 1. Introduction

Breast cancer remains a predominant global health concern, representing the most commonly diagnosed malignancy in women worldwide and a leading cause of cancer mortality [1]. In 2020, there were an estimated 2.3 million new cases of female breast cancer and 685,000 deaths globally [2,3]. This incidence accounts for about 11.7% of all new cancer cases, making breast cancer the single largest contributor to the global cancer burden [3]. Owing to improvements in detection and treatment, the number of women living with breast cancer has risen substantially and is predicted to increase to over 3 million new cases and 1 million deaths by 2040 [2], reflecting the high prevalence and survivorship of this disease.

In the United States, breast cancer is the most common cancer in women (comprising about 30% of all female cancers) and remains a major cause of cancer death [4]. Between 2012 and 2021, breast cancer incidence among U.S. women rose by approximately 1% annually, with a greater increase observed in women under 50 years (1.4% per year) compared with those 50 and older (0.7%). The sharpest rise occurred among Asian American/Pacific Islander women (2.7% and 2.5% per year in younger and older groups, respectively), whose incidence reached 86.3 per 100,000 in 2021, comparable to White women (86.4 per 100,000). Despite a 44% decline in overall mortality from 1989 to 2022, preventing about 517,900 deaths, significant racial disparities persist, with mortality remaining unchanged in American Indian/Alaska Native women and 38% higher in Black women than in White women [5].

Beyond its overall frequency, breast cancer is a clinically heterogeneous disease comprising several molecular subtypes with distinct epidemiology. Approximately 70–80% of breast tumors are hormone receptor-positive (estrogen and/or progesterone receptor-positive), corresponding to the Luminal A or Luminal B intrinsic subtypes [6,7]. In contrast, about 10–15% of cases are triple-negative (estrogen, progesterone, and HER2 receptor-negative, also called basal-like), an aggressive subtype that is disproportionately observed in younger women, in certain ethnic groups, and in BRCA1 mutation carriers [8]. The remaining fraction includes HER2-enriched tumors and other less common subgroups. These differences in subtype distribution and characteristics have important implications for prognosis and therapy, underscoring the need for precise diagnostic and monitoring tools across the spectrum of breast cancer presentations [9,10,11].

Despite progress in breast cancer outcomes over the past decades, conventional methods for diagnosis and disease monitoring have significant limitations [12,13]. Mammography is the cornerstone of breast cancer screening and has contributed to earlier detection; however, its sensitivity is reduced in women with radiologically dense breast tissue, which can lead to missed early cancers [14,15]. Moreover, mammographic screening and other imaging modalities can yield false-positive findings and overdiagnosis, prompting unnecessary invasive procedures (biopsies or surgeries) and attendant patient anxiety [16]. For patients with established breast cancer, definitive diagnosis and tumor characterization still rely on tissue biopsy of the primary tumor or metastatic sites [17,18,19]. While tissue biopsy allows histopathological assessment and molecular profiling, it is an invasive procedure that cannot be performed repeatedly with ease. Serial biopsies for monitoring are often impractical due to procedural risks and discomfort, and sampling a single lesion may not capture the full molecular heterogeneity of a patient’s cancer [20,21]. Indeed, breast cancers are typically heterogeneous, composed of multiple subclonal populations that can evolve over time and in response to therapy [22]. A single tissue sample provides only a static, partial snapshot of the disease, potentially missing resistant or aggressive subclones that exist elsewhere in the body [20]. This spatial and temporal heterogeneity can undermine the utility of tissue biopsies for guiding therapy over the course of disease. Routine clinical surveillance after initial treatment is largely based on imaging and periodic physical exams; blood tumor markers such as CA 15-3 or CEA are sometimes monitored in advanced disease, but these conventional serum biomarkers have poor sensitivity and specificity for early detection of recurrence [23]. For example, the cancer antigen 15-3 (CA15-3) assay, while widely used in monitoring metastatic breast cancer, often yields elevated levels only in a subset of patients and lacks the sensitivity needed to detect early relapse or minimal residual disease [24]. The upshot of these limitations is that metastases or disease progression may go undetected until they are radiologically apparent, and opportunities for early therapeutic intervention might be missed.

Against this backdrop, there is growing enthusiasm for liquid biopsy approaches and multi-omics biomarker strategies to transform breast cancer care. Liquid biopsy refers to the analysis of tumor-derived material circulating in body fluids (most commonly blood), including circulating tumor DNA (ctDNA), circulating tumor cells (CTCs), extracellular vesicles, and other macromolecules shed by tumor cells [17,19,20,21]. This approach offers several fundamental advantages over traditional tissue biopsy. First, liquid biopsy is minimally invasive, typically requiring a simple blood draw, and thus can be performed repeatedly with little risk or discomfort to the patient [25]. The ability to sample blood at multiple time points enables longitudinal monitoring of the tumor’s molecular status, which is critical for early detection of recurrence and real-time assessment of treatment response [25]. Second, because tumor-derived molecules in the circulation can originate from any site of disease, liquid biopsies may better capture the molecular heterogeneity of multifocal or metastatic cancers. In contrast to a single-site tissue biopsy, a blood sample can contain ctDNA fragments released from both primary and metastatic lesions, offering a more comprehensive “molecular portrait” of the disease [26]. Studies have indeed suggested that liquid biopsy can reveal genetic or epigenetic alterations present in divergent tumor subclones and can thus uncover therapeutic targets or resistance mutations that might be missed by analyzing one tissue sample [26]. Additionally, circulating biomarkers can indicate the presence of minimal residual disease (MRD) at levels far below the detection threshold of imaging, raising the possibility of earlier intervention in the relapse setting [27]. Technological advances in assay sensitivity, such as digital PCR and next-generation sequencing, now allow detection of tiny fractions of mutant ctDNA against a high background of normal DNA, making it feasible to identify one cancer molecule among thousands or even millions of normal molecules in blood.

Beyond genomics, an important paradigm shift in biomarker development is the integration of multi-omics data [28]. Breast cancer development and progression involve complex changes across the genome, epigenome, transcriptome, proteome, and metabolome. No single type of biomarker is likely sufficient to capture this complexity [29,30]. Multi-omic biomarkers refer to those that combine information from multiple molecular levels to improve diagnostic or prognostic performance. For example, in addition to genetic mutations, changes in DNA methylation patterns, gene expression (circulating tumor RNA), circulating protein markers, or even metabolites might serve as early signals of cancer presence or indicators of therapeutic response [31,32,33]. Notably, in breast cancer, DNA methylation signatures in ctDNA have emerged as especially promising early detection markers, and their power can be amplified by coupling them with other omics signals (such as fragmentomic features or protein biomarkers) [34]. Multi-omic liquid biopsy approaches thus hold the potential to detect tumoral signals that would be missed by one-dimensional tests, and to provide a more robust and comprehensive indication of tumor presence, subtype, and behavior.

This review examines recent advances in liquid biopsy and multi-omic biomarkers for breast cancer, emphasizing their role in overcoming persistent challenges in early detection, therapeutic guidance, and disease monitoring. It summarizes progress in ctDNA sequencing, CTC analysis, and emerging platforms for circulating RNA, protein, and metabolite profiling, highlighting their integration within multi-omic frameworks. Current clinical evidence demonstrates how these tools inform treatment selection, predict therapeutic response, and enable early detection of relapse or resistance. Drawing on translational research from 2019 to 2025, this review outlines how liquid biopsy and multi-omics are evolving from experimental innovation to routine clinical practice, driving more precise, personalized, and adaptive management of breast cancer.

## 2. Technological Platforms and Molecular

Liquid biopsy has revolutionized the molecular management of breast cancer, enabling non-invasive, real-time profiling of tumor dynamics that complements traditional imaging and tissue-based diagnostics. Its rapid adoption has been driven by technological advances that allow detection and characterization of circulating tumor DNA (ctDNA), cell-free DNA (cfDNA), circulating tumor cells (CTCs), exosomes carrying non-coding RNAs, and proteomic and metabolomic biomarkers. Collectively, these analytes have expanded the scope of precision oncology, offering insights into therapeutic response, resistance mechanisms, and disease evolution [35].

### 2.1. Circulating Tumor DNA (ctDNA) and Cell-Free DNA (cfDNA)

Cell-free DNA (cfDNA) consists of nucleic acid fragments released into the bloodstream during normal cell turnover, inflammation, or malignant cell death, while ctDNA represents the tumor-derived fraction that mirrors the somatic genomic landscape of cancer [36]. In breast cancer, ctDNA frequently contains driver alterations in PIK3CA, ESR1, TP53, and ERBB2, alongside copy-number changes and methylation patterns that reflect molecular subtypes [37,38].

Recent improvements in sequencing sensitivity, such as droplet digital PCR, hybrid-capture next-generation sequencing (NGS), and error-corrected sequencing, have reduced the detection threshold to variant allele frequencies below 0.01%, facilitating the identification of minimal residual disease (MRD) and early relapse [39,40,41,42]. Multi-analyte assays combining mutational, methylation, and fragmentomic data have shown superior diagnostic performance, particularly in low-shedding or early-stage tumors, where mutation-only assays are insufficient [36,41]. Methylation profiling of circulating cfDNA is particularly valuable because epigenetic alterations arise early in tumorigenesis and remain detectable at very low tumor burdens, making this approach well suited for both early cancer detection and highly sensitive minimal residual disease monitoring.

Clinically, dynamic changes in ctDNA levels have been validated as surrogate markers of therapeutic response. A decline in ctDNA following systemic therapy correlates with improved progression-free survival, whereas persistent or re-emerging ctDNA indicates resistance and impending relapse [43]. In the phase III PADA-1 trial, serial monitoring of ESR1 mutations in plasma enabled early intervention; patients switched from aromatase inhibitors to fulvestrant plus palbociclib upon detection of ESR1 mutations experienced significantly longer progression-free survival than those who continued prior therapy [44]. These findings underscore ctDNA’s potential to guide real-time therapy adjustments in hormone receptor-positive metastatic breast cancer; however, although PADA-1 demonstrated the feasibility of ctDNA-guided early switching, it has not yet established clinical utility, as neither PFS2 nor overall survival were assessed (key endpoints given the limited number of endocrine therapy options).

Beyond genomic mutations, epigenomic analysis of ctDNA is increasingly recognized for early detection and MRD monitoring. Aberrant methylation of genes such as RASSF1A, BRCA1, and SOX17 is detectable at early disease stages, often preceding radiographic changes [36,45,46]. Fragmentomic profiling, based on cfDNA fragment size, nucleosome footprints, and end motifs, further enhances sensitivity, distinguishing tumor-derived DNA from background cfDNA with machine learning–assisted classifiers [47,48].

Nevertheless, analytical challenges persist. Elevated cfDNA levels may arise from non-malignant inflammatory processes, necessitating tumor-specific variant or methylation confirmation. Moreover, clonal hematopoiesis of indeterminate potential (CHIP), particularly mutations in DNMT3A, TET2, and ASXL1, can yield false-positive results if peripheral leukocytes are not concurrently sequenced [35,49]. To mitigate these confounders, integrative bioinformatic pipelines that cross-reference methylation, fragmentation, and variant allele context are being implemented to ensure tumor-specific signal fidelity [37,50].

ctDNA now functions as both a diagnostic and pharmacodynamic biomarker. Actionable alterations identified through plasma sequencing, such as PIK3CA mutations for alpelisib eligibility, ERBB2 mutations for neratinib sensitivity, and BRCA1/2 pathogenic variants for PARP inhibition, enable non-invasive treatment selection in metastatic settings [39,51,52]. In early breast cancer, ctDNA-guided MRD surveillance is being evaluated to tailor adjuvant therapy duration and intensity, with lead times of 8–18 months before clinical relapse in pilot trials [36,53,54].

The results are supported by comprehensive clinical trials, notably the plasma MATCH study, which confirmed the capacity of ctDNA to identify actionable mutations and guide targeted therapy in patients with metastatic disease [55,56]. The integrated analysis of ctDNA and cfDNA provides a dynamic and clinically relevant perspective, bolstered by evidence that confirms their sensitivity, specificity, and practical use in tracking disease progression.

### 2.2. Circulating Tumor Cells (CTCs)

CTCs are intact malignant cells disseminated from primary or metastatic sites into the bloodstream. They serve as both prognostic and mechanistic biomarkers, reflecting metastatic potential and therapeutic responsiveness [35]. The FDA-approved CellSearch^®^ system, based on EpCAM-mediated immunomagnetic capture and cytokeratin labeling, remains the clinical reference for CTC enumeration [57]. Baseline counts ≥ 5 CTCs per 7.5 mL of blood in metastatic breast cancer correlate with inferior overall and progression-free survival, while reductions under therapy predict improved outcomes [39,58,59,60]. In early-stage disease, even a single detectable CTC increases recurrence risk and may signify subclinical metastasis [35].

To overcome the epithelial bias of EpCAM-based systems, newer microfluidic and label-free enrichment platforms capture both epithelial and mesenchymal CTCs, improving detection sensitivity in EMT-driven subtypes [37,61]. Single-cell RNA sequencing and whole-exome sequencing of CTCs have revealed significant intrapatient heterogeneity, including evolving ESR1, PIK3CA, and ERBB2 mutations that can diverge from primary tumor profiles and guide therapy selection [43,62].

Phenotypic characterization of CTCs has further clinical implications. Discordance in HER2 or estrogen receptor expression between CTCs and archival tissue can uncover candidates for targeted agents such as trastuzumab deruxtecan, even in patients previously categorized as HER2-low [44,63]. Moreover, CTC clusters (aggregates of tumor cells in circulation) exhibit unique transcriptional programs associated with stemness and immune evasion, correlating with metastatic potential and resistance to systemic therapy [39,64].

Although inter-laboratory standardization remains a challenge, emerging data indicate that integrating CTC metrics with ctDNA and imaging improves prognostic accuracy and refines risk stratification. This complementary approach leverages both genomic and cellular dimensions of tumor biology for comprehensive disease monitoring [39,65].

### 2.3. Non-Coding RNAs and Exosomes

Exosomes (nanometer-sized extracellular vesicles that reach 40–150 nm) carry stable cargoes of RNA, DNA, proteins, and lipids that reflect their cellular origin. Among their nucleic acid contents, non-coding RNAs (ncRNAs), including microRNAs (miRNAs), long non-coding RNAs (lncRNAs), and circular RNAs (circRNAs), have emerged as robust biomarkers in breast cancer [66,67,68]. These molecules regulate gene expression post-transcriptionally, orchestrating processes such as proliferation, angiogenesis, EMT, and immune evasion [69].

Clinical studies have identified several exosomal miRNAs, most notably miR-21, miR-155, and miR-10b, as elevated in breast cancer plasma, with expression levels correlating with advanced stage, chemoresistance, and unfavorable prognosis [66,70,71]. Similarly, oncogenic lncRNAs such as HOTAIR and HAGLROS are overexpressed in breast cancer exosomes. Meng et al. (2024) demonstrated that exosomal HAGLROS induces macrophage M2 polarization via the miR-135b-3p/COL10A1 axis, promoting EMT and angiogenesis, and ultimately driving tumor progression [69].

Technological refinements in exosome isolation, including size-exclusion chromatography and immunoaffinity capture targeting tetraspanins (CD9, CD63, CD81), have improved reproducibility but require harmonization for clinical use [35,72,73]. Quantitative analysis of exosomal ncRNAs through digital PCR and next-generation sequencing has allowed the development of machine-learning classifiers that combine multiple ncRNAs for enhanced diagnostic accuracy. Cardinali et al. (2022) reported that miRNA panels integrated with clinical parameters achieved >85% sensitivity and >90% specificity for distinguishing breast cancer patients from healthy controls [66].

Beyond diagnostics, ncRNA dynamics offer prognostic and therapeutic insight. Downregulation of miR-21 or miR-155 following chemotherapy correlates with improved response, while persistent elevation predicts residual disease [66,74,75]. Furthermore, ncRNA cargo reflects immune-tumor interactions, and emerging evidence suggests exosomal ncRNA profiling could serve as a biomarker for immune checkpoint inhibitor efficacy. The stability of exosomal RNA in biofluids makes it an ideal substrate for serial sampling in longitudinal monitoring of treatment efficacy and disease progression [69,76].

### 2.4. Proteomic and Metabolomic Signatures

Beyond nucleic acids, proteomic and metabolomic profiling from liquid biopsies provide functional layers that capture tumor metabolism and microenvironmental adaptation. Mass spectrometry-based metabolomics offer complementary insights into protein expression and metabolic pathway reprogramming associated with breast cancer progression [37,77].

Data-independent acquisition (DIA) mass spectrometry, tandem mass tag (TMT) labeling, and aptamer-based proteomic arrays now enable high-throughput quantification of circulating and extracellular vesicle (EV)-associated proteins [78]. In a 2024 study, Xu et al. identified serum EV proteins such as TALDO1 as potential biomarkers for distant metastasis; inhibition of TALDO1 suppressed invasion and metastasis in preclinical models, underscoring the translational potential of EV proteomics [79]. Functional proteomic signatures have also been correlated with therapeutic response and metastatic risk, complementing ctDNA data for real-time disease monitoring [37].

Metabolomic studies reveal characteristic alterations in amino acid, lipid, and nucleotide metabolism reflecting tumor hypermetabolism. Elevated lactate, choline derivatives, and TCA-cycle intermediates mirror bioenergetic rewiring and proliferation. Integrated multi-omic models that combine proteomic, metabolomic, and ctDNA data achieve higher diagnostic accuracy than any single modality alone [36,80]. Moreover, specific metabolic signatures, such as activation of the pentose phosphate pathway and aberrant lipid oxidation, are being explored as therapeutic vulnerabilities [79].

**Figure 1:**
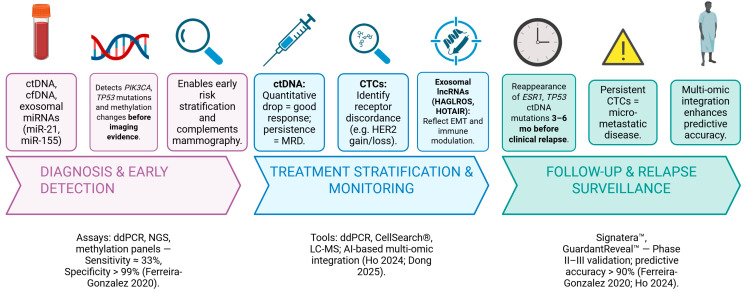
Integrated role of liquid biopsy across diagnosis, treatment, and follow-up in breast cancer. During diagnosis and early detection, circulating tumor DNA (ctDNA), cell-free DNA (cfDNA), and exosomal microRNAs enable early molecular identification of mutations and methylation changes that may precede imaging findings. In the treatment phase, quantitative ctDNA monitoring reflects therapeutic response and minimal residual disease, while circulating tumor cells (CTCs) and exosomal long non-coding RNAs provide insights into receptor discordance, epithelial–mesenchymal transition, and immune modulation. For follow-up and relapse surveillance, the reappearance of ESR1 or TP53 mutations and persistent CTCs serve as early indicators of recurrence or micrometastatic disease. Multi-omic integration and AI-based analytical tools further enhance the predictive accuracy of these biomarkers, supporting personalized and adaptive management strategies in breast cancer [37,39,84].

Standardization remains a key obstacle to clinical translation. Variability in pre-analytical handling, storage, and instrument calibration can affect reproducibility, underscoring the need for consensus protocols. Nevertheless, the integration of advanced mass spectrometry with artificial intelligence (AI)-driven pattern recognition promises scalable, automated pipelines for multi-omic biomarker discovery (Figure 1) [36,81,82,83].

Table 1 provides an overview of leading commercial liquid biopsy assays, outlining their analytical foundations, detection capabilities (including CNVs, RNA, and gene fusions), and technical sensitivity thresholds. The platforms span broad genomic profiling, ctDNA-based minimal residual disease detection, multi-cancer methylation screening, and emerging cfRNA technologies. The assays listed, including Guardant360 CDx [85], FoundationOne Liquid CDx [86], Signatera [87], TruSight Oncology 500 ctDNA [88], Oncomine cfDNA/cfRNA [89], cobas EGFR Mutation Test v2 [90], and RARE-seq [91], cover the spectrum from broad genomic profiling to highly sensitive minimal residual disease detection and emerging cfRNA technologies.

## 3. Clinical Applications Across the Continuum of Care

### 3.1. Early Detection and Screening

Earlier detection of breast cancer improves the likelihood of cure because tumors diagnosed at lower stage are more amenable to definitive local therapy and have greater sensitivity to systemic treatment [4]. Conventional population screening relies primarily on digital mammography, which reduces mortality but has decreased performance in radiographically dense breasts and can generate false positives that lead to unnecessary procedures [15]. Adjunct imaging with ultrasound or MRI improves detection in dense tissue but increases cost and resource demands, motivating interest in blood-based tests that could complement imaging without added radiation or prolonged appointments [14,15].

Liquid biopsy approaches are advancing as potential adjuncts to screening by detecting tumor-derived signals in plasma before radiographic abnormalities are apparent [43]. Among circulating analytes, cell-free DNA (cfDNA) methylation profiling has emerged as a particularly sensitive readout because epigenetic alterations occur early and are abundant across the genome, enabling detection in low-shedding tumors where mutation-only assays underperform [36,92]. Plasma-only methylome and fragmentome models that integrate methylation, size profiles, and nucleosome features have achieved substantially higher sensitivity than mutation-only assays in early-stage disease while maintaining high specificity in controls [36,93]. Although multi-cancer early detection blood tests are not yet approved for population screening, proof-of-concept studies indicate that a composite multi-omic plasma signature could flag asymptomatic cancers and guide targeted imaging, a paradigm now being explored prospectively in breast cancer–enriched cohorts [20,94,95].

Multi-omic risk-adapted screening models that fuse germline risk, clinical factors, and circulating biomarkers are under development to individualize starting age, frequency, and modality of screening, with the goal of increasing detection of biologically aggressive disease while minimizing over-diagnosis and false positives [20,96,97,98]. As these platforms mature, the near-term clinical path is likely an adjunct strategy in which abnormal blood signals triage individuals to diagnostic imaging or shortened follow-up intervals rather than replace mammography outright [43,99].

### 3.2. Minimal Residual Disease and Relapse Monitoring

Minimal residual disease (MRD) denotes the presence of molecular evidence of cancer during apparent clinical remission and is a major driver of late recurrences in early-stage breast cancer [35,54]. Traditional surveillance with clinical examination, imaging, and nonspecific serum markers such as CA15-3 has limited sensitivity for subclinical relapse, frequently detecting recurrence only after macroscopic disease emerges [35,100]. Circulating tumor DNA (ctDNA) has transformed MRD assessment by enabling detection of tumor-specific alterations months before radiographic recurrence, opening a therapeutic window for preemptive intervention [36]. Personalized, plasma-only assays that combine tumor-informed mutations with methylation or fragmentomics have reported lead times approaching one to one-and-a-half years between molecular relapse and clinical detection, with ctDNA negativity strongly associated with durable remission [36,101,102].

In the neoadjuvant setting, integrated analyses show that early ctDNA clearance correlates with pathologic complete response, whereas persistent ctDNA after chemotherapy identifies patients with residual viable disease biology who are at heightened risk for relapse [37]. Dong and colleagues combined tumor genomics, transcriptomics, and serial ctDNA to show that molecular residual disease signatures outperformed imaging alone for predicting recurrence after neoadjuvant therapy, supporting escalation or switch strategies guided by ctDNA status [37]. Complementary markers such as circulating tumor cells (CTCs) and extracellular vesicle (EV) cargo provide additional biological context, but for MRD detection the sensitivity of ctDNA remains superior because viable CTCs are rare in early-stage disease and isolation methods can miss mesenchymal phenotypes [39,103,104].

Prospective interventional trials are now testing whether acting on ctDNA positivity improves outcomes, by intensifying systemic therapy, initiating targeted agents, or triggering earlier imaging, while carefully balancing the risks of overtreatment and patient anxiety [43,105,106,107]. Standardization of pre-analytical handling, sequencing, and bioinformatic pipelines and harmonized reporting thresholds remain prerequisites for broad adoption; current professional guidance recommends analytically validated assays, leukocyte sequencing to mitigate clonal hematopoiesis, and serial testing to confirm molecular relapse before clinical action [23].

### 3.3. Therapy Response Prediction

Dynamic molecular monitoring enables prediction of therapeutic response and early detection of resistance, allowing treatment adaptation before overt progression [43,104]. Early declines in ctDNA variant allele fractions within weeks of starting therapy correlate with radiographic response and longer progression-free survival across systemic regimens, whereas persistent or rising ctDNA predicts primary resistance and impending progression [39,108]. In ER-positive/HER2-negative metastatic breast cancer, the phase III PADA-1 trial demonstrated the potential clinical utility of serial ctDNA: patients with emergent ESR1 mutations during aromatase inhibition who switched early to fulvestrant plus palbociclib had significantly longer progression-free survival than those who continued the aromatase inhibitor, validating ctDNA-triggered therapy redirection [44,109,110].

The SERENA-6 trial (NCT04964934) is also a major ongoing study evaluating ctDNA-guided endocrine therapy adaptation. This double-blind, phase III trial tests whether switching from an aromatase inhibitor to the next-generation oral SERD camizestrant upon detection of an ESR1 mutation in ctDNA can delay clinical progression in HR+/HER2– advanced breast cancer. While SERENA-6 is an important step in validating molecularly driven early switching, it does not yet establish clinical utility for reasons similar to PADA-1: long-term endpoints such as PFS2 and overall survival remain unreported, and only approximately 10% of screened patients were eligible for randomization, raising both scientific and ethical challenges regarding generalizability. Early-phase data from SERENA-1 have shown that camizestrant is well tolerated and demonstrates antitumor activity across multiple dose levels, including in heavily pre-treated patients and those with ESR1-mutated disease, supporting the biological rationale for SERENA-6 but not yet confirming a change in clinical practice [111,112].

Actionable ctDNA findings routinely guide therapy selection in advanced disease: PIK3CA mutations identify candidates for PI3Kα inhibition, activating ERBB2 mutations support HER2-targeted kinase therapy, and pathogenic BRCA1/2 alterations inform PARP inhibitor sensitivity, with plasma genotyping providing a non-invasive alternative when tissue is unavailable or inadequate [39,57,113]. In the neoadjuvant setting, multi-omic modeling that integrates pretreatment tumor expression programs and early ctDNA kinetics stratifies probability of pathologic complete response, enabling de-escalation for likely responders and early switch for non-responders [37].

CTC assays add a complementary cellular dimension: enumeration at the threshold of ≥5 CTCs/7.5 mL blood in metastatic disease is associated with inferior outcomes, and on-treatment reductions correlate with benefit, while phenotypic characterization can reveal discordant hormone receptor or HER2 status relative to archival tissue that may open targeted options such as antibody–drug conjugates [39,57,59]. Emerging single-cell multi-omic analyses of CTCs uncover resistance programs, such as ESR1 ligand-binding domain mutations, PI3K pathway activation, and EMT-linked transcriptomes, that can be therapeutically actionable or prognostic, though standardized workflows and prospective validation are still needed for routine decision-making [43,114,115].

Exosome-associated non-coding RNAs (ncRNAs) are increasingly studied as dynamic biomarkers of treatment response and immune modulation; for example, elevated circulating miR-21 and miR-155 associate with chemoresistance and endocrine resistance, and changes in their levels during therapy track with response in multiple cohorts [66,116,117,118]. Studies show that the lncRNA HAGLROS packaged in tumor-derived exosomes promotes M2 macrophage polarization and EMT via the miR-135b-3p/COL10A1 axis, providing a plausible link between EV-ncRNA profiles and resistance phenotypes in the tumor microenvironment [69,119]. Proteomic biomarkers extend this functional readout: EV-proteomic signatures identify patients at risk of early metastasis, and targeting the pentose phosphate pathway enzyme TALDO1, nominated by EV-proteomics, suppressed invasion and metastasis in preclinical models, illustrating the theranostic potential of proteomic discovery [79].

The future of response prediction is decisively multi-omic: models that integrate ctDNA mutations and methylation, tumor transcriptomics, EV-proteins, and metabolomics consistently outperform single-omic approaches and are being advanced into prospective trials with embedded clinical decision support [20,37,120]. Implementation will require harmonized assay standards, equitable access, and prospective thresholds that balance sensitivity with actionability to avoid premature therapy changes [23].

### 3.4. Companion Diagnostics

Companion diagnostics (CDx) are analytically and clinically validated tests that identify patients likely to benefit from a specific therapy and are central to precision oncology in breast cancer [23,121,122]. Tissue-based ER/PR immunohistochemistry and HER2 IHC/ISH remain foundational CDx that direct endocrine therapy and HER2-targeted treatment and have transformed outcomes across disease stages [4,123]. Plasma-based CDx now complement tissue testing: ctDNA assays detecting PIK3CA mutations can determine eligibility for PI3Kα inhibitors when tissue is inaccessible or archival results are discordant, and ESR1 mutation testing can guide selection of selective estrogen receptor degraders after aromatase inhibitor exposure, exemplifying the evolution from static baseline characterization to dynamic, longitudinal eligibility assessment [39,84,124].

Professional recommendations emphasize that CDx, whether tissue or plasma, must meet rigorous criteria for analytical validity, clinical validity, and clinical utility, including demonstrated benefit when therapy is selected based on the test result, and that ctDNA assays used for CDx should include controls for clonal hematopoiesis and report assay limits of detection and confidence intervals for negative results [23,86,125,126]. CTC-based CDx are an area of active development: HER2 expression on CTCs may identify candidates for HER2-directed agents despite HER2-negative primary tumors, but assay heterogeneity and epithelial marker bias have limited regulatory adoption to date, spurring development of EpCAM-independent capture and standardized readouts [39,127,128].

Emerging CDx modalities extend beyond genomics. Exosomal ncRNA panels that track resistance pathways, such as miR-21/miR-155 or HAGLROS-driven macrophage polarization, are being evaluated as predictors of endocrine or chemotherapy benefit, and EV-proteomic signals such as TALDO1 illustrate how a biomarker can be both diagnostic and a drug target, aligning with a theranostic paradigm [66,129,130]. As multi-omic evidence accrues, adaptive CDx that dynamically update treatment eligibility based on serial ctDNA, CTC, and EV-omic profiles are likely to underpin umbrella and platform trials and to be embedded in electronic decision support for real-time care (Table 2) [20].

## 4. Multi-Omic Integration for Precision Oncology

High-throughput molecular technologies now enable comprehensive characterization of tumors across multiple “omics” layers, including genomics, transcriptomics, proteomics, and metabolomics. Integrating these diverse data types (a strategy known as multi-omic integration) provides a holistic view of cancer biology, beyond what any single omic could reveal [132]. In precision oncology, such integrated analyses can identify molecular drivers and vulnerabilities unique to each patient’s tumor, paving the way for highly personalized treatments [132,133,134]. Effective multi-omics integration requires sophisticated computational approaches to handle the sheer volume and complexity of data. Recent methods range from unsupervised algorithms for pattern discovery to supervised machine learning models that correlate multi-omic features with clinical outcomes [135,136,137]. This section reviews how different omics synergize in cancer, the role of artificial intelligence (AI) in multi-omic biomarker integration, and examples of multi-omics guiding clinical oncology.

### 4.1. Synergy of Genomics, Transcriptomics, Proteomics, and Metabolomics

Each omic layer captures a distinct aspect of tumor biology, and their integration yields a more complete molecular portrait of cancer. Genomics profiles DNA-level alterations (mutations, copy-number changes, etc.) that can drive cancer and inform targeted therapies. Transcriptomics measures gene expression, reflecting which genes are actively transcribed and highlighting pathways or fusions not evident from DNA alone [131,138,139]. Notably, genomic and transcriptomic analyses are often complementary. Studies have found that many driver genes or biomarkers are identified by one modality but not the other [140,141,142]. This complementarity underscores the need to combine multiple omics to avoid missing critical signals. Proteomics adds another layer by quantifying protein expression and modifications. Because post-transcriptional regulation can lead to discordance between mRNA and protein levels, proteomic data often reveal functional changes (e.g., activated signaling proteins or phosphorylated enzymes) that genomics/transcriptomics might not predict. Integrating proteogenomic data has, for example, uncovered new tumor subtypes and therapeutic targets by connecting genetic alterations to their downstream protein effects [143,144,145,146,147]. Metabolomics, meanwhile, captures the metabolic state of cancer cells and the tumor microenvironment. Metabolic reprogramming is a hallmark of cancer, and profiling metabolites can highlight pathway dependencies or immune–metabolic interactions that are invisible to genome or proteome analysis [148,149,150]. By jointly analyzing these layers, researchers can link genotype to phenotype; for instance, seeing how a DNA mutation alters RNA and protein networks, which in turn reshapes cellular metabolism [132,151].

Unlike single-omic approaches that capture only a fragment of tumor biology, multi-omic integration overcomes these limitations by merging complementary molecular layers to generate a more complete and clinically informative picture of disease.

Multi-omics integration has fundamentally shifted cancer classification and biomarker discovery. Instead of classifying tumors solely by tissue of origin, integrated molecular profiles enable pan-cancer taxonomies based on underlying biology [132,152,153]. For example, The Cancer Genome Atlas (TCGA) compiled multi-omic data for thousands of tumors and revealed molecular subtypes that cut across traditional cancer types [154,155,156]. Pan-cancer analyses integrating genomic, epigenomic, transcriptomic, and proteomic layers have enabled the reclassification of tumors and the discovery of subgroup-specific therapeutic vulnerabilities. Foundational deep-learning frameworks capable of fusing heterogeneous multi-omic datasets, such as the pan-cancer integration model described by Zhang et al. [157], further demonstrate the computational strategies that support the development of more advanced, breast cancer–focused predictive systems.

Multi-omic approaches also capture tumor heterogeneity: within a single tumor, different regions or cell populations may harbor distinct genetic changes, expression programs, and metabolic profiles. Integrating these data can illuminate intra-tumoral heterogeneity and tumor–microenvironment interactions (such as immune infiltrate characteristics or microbiome influences) that impact disease progression and treatment response [131,139]. In short, genomics, transcriptomics, proteomics, and metabolomics each provide complementary insights, and their synergy offers an unprecedented 3D view of the “cancer landscape” that surpasses a one-dimensional map [158].

### 4.2. AI and Machine Learning in Multi-Omic Biomarker Integration

Making sense of high-dimensional multi-omic data is a major challenge. This is where artificial intelligence (AI) and machine learning (ML) play a main role. ML algorithms can integrate diverse data types to detect patterns or predictive features that would elude manual analysis [159,160,161,162,163]. A key step in multi-omic integration is dimensionality reduction or feature selection, often achieved with unsupervised methods. For instance, techniques like joint non-negative matrix factorization (jNMF) or multi-omics factor analysis (MOFA) decompose heterogeneous omic datasets into a few latent factors, clustering tumors into subgroups with shared multi-omic signatures [164,165]. Such multi-omics machineing has been shown to outperform single-omic analyses in identifying biologically relevant tumor subtypes. A recent study exemplifies the integration of artificial intelligence and multi-omic data for refined breast cancer stratification. Using transcriptomic profiles from multiple datasets, researchers developed a machine learning–based Breast Cancer Classifier (BCC) that identified a distinct HER2-low molecular subtype not captured by conventional PAM50 or immunohistochemistry assays [166]. The BCC leveraged 63 gene features to delineate HER2-low tumors with high accuracy, revealing hybrid basal–luminal expression patterns and recurrent ERBB2, EGFR, and PI3K pathway alterations. Validation across independent cohorts confirmed robust classification performance (F1 scores > 0.9) and distinct prognostic behavior, demonstrating how AI-driven transcriptomic modeling can uncover clinically relevant subgroups and enhance precision oncology through multi-omic biomarker integration [166].

Beyond unsupervised discovery, supervised ML and AI models are increasingly used to build multi-omic predictors for clinical endpoints. By training on patient datasets with known outcomes, these models learn to combine omic features into biomarkers for diagnosis, prognosis, or drug response. For example, multi-omic survival prediction networks (e.g., the SALMON deep learning model [167]) aggregate genomic and transcriptomic data to predict patient survival better than models using a single data type [157,168].

Deep learning frameworks are increasingly able to integrate heterogeneous omic layers, capturing nonlinear relationships across genomic, transcriptomic, proteomic, and metabolomic data to substantially enhance predictive accuracy and support more precise therapeutic outcome modeling.

In the study by Walbaum et al. (2025) [169], the authors analyzed 658 patients with early-stage, ER-positive/HER2-negative breast cancer stratified by age (≤40, 41–50, >50), comparing clinicopathologic and genomic signatures (PAM50/Prosigna) to develop age-specific prognostic insight. They found that young women had more aggressive tumor biology, poorer outcomes, and more frequent discordance between conventional markers (e.g., Ki67) and genomic risk scores; for instance, 50% of women ≤ 40 with Ki67 < 10% were classified as high-risk by the PAM50 risk-of-relapse (ROR) score, whereas <5% of older women showed that discordance. The genomic ROR signature remained independently prognostic within the younger group, outperforming standard clinical markers, thus illustrating how integrating genomic expression profiles with clinical context (here, age stratification) yields more accurate risk stratification than clinical features alone.

Sharma and colleagues conducted a multi-omic integration analysis of breast cancer tissues from 335 patients in the long-term Oslo2 cohort (>12 years follow-up), applying MOFA+ to jointly analyze transcriptomic, proteomic, and metabolomic data alongside clinical information, and derived three distinct prognostic clusters with significantly different survival (*p* = 0.005) [170]. These clusters outperformed conventional intrinsic subtypes (such as PAM50) in long-term outcome prediction, including in external validation using METABRIC and TCGA cohorts, demonstrating that combining molecular layers (gene expression, protein, metabolite) plus clinical features can stratify patients into prognostic groups more precisely than any single modality alone [170]. The multi-omic clusters also showed differential enrichment in cell-cycle- and immune-related pathways, underscoring the biologic interpretability of the integrative model beyond black-box risk scores [170].

In the realm of therapy selection, machine learning has identified multi-omic signatures that predict drug sensitivity or resistance. These include composite biomarkers that may involve a DNA mutation plus an expression level and a protein activation status—patterns that AI can detect across complex datasets [159,171].

Advanced AI techniques, including deep learning and ensemble methods, are especially powerful for multi-omic integration. For instance, DeepCNA utilizes advanced deep neural architectures to deduce phenotypes associated with copy number variations from highly fragmented circulating free DNA (cfDNA), thereby enhancing the characterization of subclonal tumor populations within a heterogeneous disease context [172]. Neural networks can be architected with multiple input branches, where each branch processes one omic data type before a late integration layer fuses the learned representations [157,168,173,174]. This design allows the model to handle heterogeneous data (for example, mutations, gene expression profiles, and proteomic spectra) in parallel, preserving the specific structure of each omic. Such deep models have successfully identified diagnostic and predictive biomarker combinations across oncology and other fields [175,176]. Recent developments even incorporate non-traditional data streams. For instance, integrating radiomics (imaging features) [177,178] or digital pathology with genomic/proteomic data [179,180] further enriches prediction models, though those extend beyond the classic “four omics”.

Key advantages of AI-driven multi-omic integration include the ability to detect higher-order interactions (e.g., a gene mutation’s effect may only be evident when considering a correlating metabolite level) and to accommodate the sheer scale of features (tens of thousands of measurements per tumor) [181,182]. For example, machine learning approaches have identified multi-omic biomarker panels for early cancer detection and for predicting immunotherapy response, outperforming traditional single-marker assays in some studies [183,184,185].

Nonetheless, applying AI to multi-omics is not without challenges. Models can be prone to overfitting given the high feature-to-sample ratio typical of omics studies, and ensuring interpretability is difficult when complex algorithms combine data in nonlinear ways [186,187]. There is growing emphasis on explainable AI techniques to decipher which features drive a prediction (e.g., which genes or proteins are most influential in a prognostic model) so that the results can be trusted by clinicians [188,189,190]. Moreover, integrating disparate data types requires careful normalization and handling of batch effects. Despite these hurdles, the trend is clear: AI and machine learning are invaluable for merging multi-omic inputs into actionable cancer biomarkers, and their role will only expand as multi-omic datasets grow larger and more intricate [185,190]. However, it is important to clarify that most AI-driven multi-omic models remain in the research or early validation phase and have not yet received regulatory approval for routine clinical use.

### 4.3. Evidence from Clinical Studies

The promise of multi-omic integration is now being tested in clinical research and trials, with encouraging results. Large-scale consortia have already used multi-omics to generate biological insights with clinical implications. For instance, as mentioned above, the TCGA program’s pan-cancer multi-omic dataset has been mined to identify new tumor subtypes and biomarkers, some of which have informed updates to cancer classifications and potential therapeutic targets [154,155].

An increasing body of clinical research demonstrates that the integration of multi-omic data, driven by artificial intelligence (AI) and machine learning (ML), is transforming complex molecular information into clinically actionable tools for precision management of breast cancer. For example, Sammut et al. (2022) integrated genomic, transcriptomic, and immune-microenvironment features from pretreatment biopsies in 168 breast cancer patients receiving neoadjuvant therapy, demonstrating that ML models combining these data predicted pathological complete response with an AUC ≈ 0.87, outperforming conventional clinicopathologic factors [191]. Similarly, Dong et al. (2025) used a multi-omic framework linking ctDNA dynamics, tumor transcriptomics, and proteomic data to predict recurrence after neoadjuvant chemotherapy; integrated molecular residual disease signatures more accurately forecasted relapse risk than imaging alone [37].

Other studies have advanced prognostic and therapeutic stratification through large-scale, AI-driven integration of diverse molecular layers. Liu et al. (2025) developed an immune-related long non-coding RNA signature by testing over one hundred ML model combinations on transcriptomic data; the resulting nine-lncRNA score (IRLS) stratified survival and chemotherapy response across seventeen independent cohorts, surpassing 95 existing prognostic models [192]. Sathyamoorthi et al. (2025) combined genomic, epigenomic, transcriptomic, and clinical features from TCGA to train a deep-learning survival model, improving predictive accuracy by approximately 12% over single-omic inputs [193]. Zhang et al. (2024) similarly demonstrated that a deep multi-omics fusion network integrating copy-number variation, gene expression, and clinical data achieved an AUC ≈ 0.99 for survival prediction, illustrating the potential of neural-network architectures to exploit high-dimensional biomolecular data [194].

Multi-omics has also refined molecular classification and early detection. Omran et al. (2025) integrated transcriptomic, DNA-methylation, and microbiome profiles from 960 breast tumors using the MOFA+ algorithm, identifying latent molecular factors that enhanced PAM50 subtype discrimination and revealed immune and vesicle-trafficking pathways linked to subtype biology [195]. Complementing these tissue-based studies, An et al. (2022) combined plasma metabolomics and proteomics in 216 women with breast cancer or benign disease to identify amino-acid and redox-pathway signatures distinguishing malignancy with AUCs of 0.79–0.88, demonstrating the feasibility of integrative liquid-biopsy screening [196]. Collectively, these studies underscore the clinical potential of multi-omic and AI-based approaches to improve early detection, therapy guidance, and survival prediction in breast cancer.

## 5. Challenges, Limitations, and Ethical Considerations

Despite the excitement, it is important to acknowledge that routine clinical adoption of multi-omic integration is still in its early stages. Practical challenges remain before this approach becomes standard-of-care [135]. One major issue is data management and analysis: generating terabytes of multi-omic data is now technically possible, but interpreting that data for an individual patient in real time requires advanced bioinformatics pipelines and expert teams [197,198]. Standardization is another hurdle: different platforms and assays need harmonization, and results must be reproducible across laboratories. Additionally, the regulatory and reimbursement environment for multi-omic tests is complex [199,200]. Unlike single-gene tests, multi-omic assays face uncertain paths for approval and insurance coverage. Regulators will demand evidence that multi-omic decision-making improves outcomes before endorsing widespread use, which means more clinical trials and longitudinal studies are needed [199,201,202]. Data interpretation and clinical interpretability are also concerns—physicians need user-friendly reports that distill multi-omic data into clear treatment recommendations, often aided by AI-based decision support [203].

### 5.1. Technical and Biological Hurdles in Breast Cancer Biomarkers

Despite significant advances in breast cancer biomarkers, numerous technical hurdles impede their reliable integration into practice. A major challenge is ensuring reproducibility and standardization of biomarker assays across different laboratories. Variability in sample handling, assay methods, and interpretation can lead to inconsistent results. For example, early proteomic biomarker studies using SELDI-TOF mass spectrometry suffered from poor reproducibility due to low resolution and chip-to-chip variation, whereas more advanced MALDI-TOF-MS techniques improved robustness but still remained sensitive to impurities, affecting result consistency [204]. Similarly, variations in reagent chemistry or data analysis pipelines can yield irreproducible findings if protocols are not standardized [205]. These issues underscore the need for rigorous assay validation, quality control, and inter-laboratory proficiency testing for any proposed biomarker test [206,207]. Even for well-established biomarkers like HER2, studies have noted that differences in pre-analytic tissue processing, choice of antibodies or probes, and observer interpretation can cause discrepancies in HER2 status determination [208]. Consequently, organizations such as ASCO/CAP have issued detailed testing guidelines to improve accuracy, yet achieving global consistency remains difficult [209,210,211]. Another technical hurdle is the sensitivity and limits of detection for certain biomarker modalities. Emerging “liquid biopsy” markers (e.g., circulating tumor DNA or tumor cells) often exist at very low concentrations, pushing the limits of current detection technology [204,212]. Insufficient sensitivity or specificity in these assays can lead to false negatives or false positives, undermining clinical utility.

Beyond technical issues, the inherent biological complexity of breast cancer poses substantial challenges for biomarker development. Breast tumors are highly heterogeneous diseases at the genomic, transcriptomic, and cellular levels [213]. This heterogeneity exists not only between patients (intertumor differences) but also within different regions of the same tumor (intratumor heterogeneity) and between primary and metastatic sites [214].

### 5.2. Cost and Access Barriers

Even when robust biomarkers are available, economic and access issues can limit their impact, especially in the context of breast cancer [215]. The development and deployment of advanced biomarker tests (such as multi-gene expression panels or next-generation sequencing) can be expensive [216]. Many high-profile breast cancer assays, for example, the 21-gene Oncotype DX recurrence score test used to guide adjuvant therapy, carry substantial costs (on the order of several thousand USD per test) that may not be affordable or reimbursed in all healthcare systems. Indeed, studies have shown that while tests like Oncotype DX can be cost-effective in preventing unnecessary chemotherapy in the long run, the upfront cost remains a barrier in some settings [217,218]. For instance, access to life-saving anti-HER2 therapy requires reliable HER2 testing, yet surveys indicate that in some low- and middle-income countries a significant fraction of breast cancer patients do not receive HER2 testing due to lack of pathology infrastructure and cost constraints [219]. These deficiencies mean patients in those regions might not be identified for HER2-targeted treatments, exacerbating outcome disparities.

Even within wealthy countries, socioeconomic and geographic disparities in biomarker use have been documented. Snow et al. (2024) identified several factors limiting equitable access to molecular testing in cancer care across Canada. The most significant barrier is the lack of consistent public funding for genomic assays, which forces many patients to rely on clinical trials or cover the costs themselves [220].

Geographic and infrastructural disparities also play a major role, as rural and remote areas often lack specialized laboratories, leading to delays and compromised sample quality. Additional challenges include limited clinician awareness or willingness to request molecular tests, as well as inconsistencies in turnaround times and reporting formats that hinder the timely application of results. At the patient level, the authors highlight barriers such as low health literacy, mistrust of genomic medicine, and socioeconomic constraints that restrict access to precision diagnostics and personalized treatment [220].

Another population-based study, based in the U.S., found that only about 38% of eligible women with early-stage breast cancer received the Oncotype DX genomic test, and the likelihood of getting tested was significantly lower for certain groups. Black women, for example, had testing rates around 31%, markedly below the rate in non-Black women. Low-income patients were also less likely to be tested (only 24% uptake in the lowest income group) despite being eligible [221]. These gaps suggest that cost and healthcare access issues, such as insurance coverage, patient out-of-pocket costs, and availability of testing facilities, influence who benefits from precision diagnostics.

Another critical consideration is the perceived cost vs. value of biomarker testing. Payers have sometimes been hesitant to cover expensive tests without clear evidence of cost-effectiveness. However, recent studies provide reassuring data: the integration of broad molecular testing does not necessarily increase overall treatment costs and can even be cost-saving by optimizing therapy [222]. For instance, a 2025 study showed that patients who underwent comprehensive genomic profiling did not incur higher total healthcare costs in first-line treatment compared to those who received more limited or no testing [223].

### 5.3. Ethical Issues in Biomarker Use

The rise of precision biomarkers in breast cancer brings not only clinical benefits but also a host of ethical considerations. One major concern is patient privacy and data security. Many biomarker tests, especially genomic assays, generate sensitive genetic information. Patients and professionals worry that these data could be misused if they fall into the wrong hands—for example, by health insurers or employers to discriminate against individuals with certain genetic risks [224]. Although laws in some countries (e.g., GINA in the U.S.) restrict genetic discrimination in health insurance, gaps remain (such as life or disability insurance, or in employment settings) [225].

Regarding this issue, Erdmann et al. (2021) analyzed the main ethical challenges associated with the use of biomarkers and genetic data in precision medicine [224]. The authors identified key concerns related to privacy, informed consent, and data ownership, noting that large-scale genomic data collection increases the risk of re-identification and misuse. They emphasized the ethical tension between promoting data sharing for research and protecting individual autonomy. The study also highlights risks of discrimination, particularly in employment or insurance contexts, and the difficulties of managing incidental findings that may have implications for patients and relatives. Finally, the authors stress that unequal access to biomarker-based technologies could worsen existing disparities in healthcare [224].

## 6. Future Perspectives and Clinical Translation

As liquid biopsy technologies advance in breast cancer, several challenges and opportunities lie ahead to translate multi-omic biomarkers into routine care. Key priorities include establishing standardization and validation of multi-omic platforms, effectively integrating liquid biopsies into clinical workflows, and developing personalized screening and dynamic monitoring models that leverage the rich data from circulating biomarkers. Below, we discuss each of these areas in an academic context, focusing on breast cancer.

### 6.1. Standardization and Validation of Platforms

A foremost challenge in bringing multi-omic liquid biopsy to the clinic is the lack of standardization across technologies and laboratories. Currently, different studies and centers employ varying methods for sample collection, processing, and analysis of circulating tumor DNA (ctDNA), circulating tumor cells (CTCs), and other analytes [20,226,227]. Pre-analytical variables such as blood draw timing, tube type, and DNA isolation protocols can significantly impact results, yet consensus protocols are still emerging. Without uniform procedures, results may vary widely between laboratories [20,228].

To address this, the field is moving toward consensus guidelines and rigorous validation of platforms. The International Society of Liquid Biopsy (ISLB) has convened expert panels to define minimal technical requirements for ctDNA assays. Standardized quality criteria must be clearly defined and universally implemented to facilitate broader clinical adoption [207]. Key considerations include appropriate blood collection methods, efficient and reproducible isolation of cfDNA, robust assay validation on large cohorts, and precise bioinformatic data interpretation [207].

Multi-omic approaches could improve diagnostic accuracy but also introduce new variability. Therefore, standardization efforts must extend beyond a single platform to integrated multi-omic workflows. Each component of a multi-omic test (for instance, a combined ctDNA mutation and methylation assay) requires validation. The lack of standardization across different LB platforms and the need for clear demonstration of clinical utility are critical factors that determine whether widespread implementation succeeds [229].

Addressing current limitations requires alignment with emerging international standardization frameworks designed to harmonize liquid biopsy methodologies. Initiatives such as the Blood Profiling Atlas Consortium (BloodPAC) have established shared data resources, consensus protocols for pre-analytical handling, and performance benchmarks to improve inter-laboratory comparability and support regulatory readiness [126]. Similarly, the European Society for Medical Oncology (ESMO) has issued technical and clinical reporting guidelines for circulating tumor DNA (ctDNA) analysis, including assay validation, quality control, and sensitivity documentation [23]. The European Liquid Biopsy Society (ELBS) and the College of American Pathologists (CAP) have launched programs to develop reference materials and external proficiency testing for ctDNA, circulating tumor cells (CTCs), and extracellular vesicle assays [230]. These collaborative efforts aim to ensure that results from different laboratories are analytically equivalent and clinically interpretable. Integrating these standards into multi-omic workflows is essential for enhancing reproducibility, facilitating clinical implementation, and supporting future regulatory evaluations of liquid biopsy technologies.

### 6.2. Integration into Clinical Workflows

Even as reliable multi-omic assays become available, a major hurdle is integrating these tools smoothly into everyday clinical decision-making for breast cancer. Oncologists and care teams are accustomed to established diagnostics (imaging, tissue biopsy, immunohistochemistry, etc.), so adding liquid biopsy requires demonstrable clinical utility and workflow adaptation. In fact, experts note that the integration of liquid biopsy tests into the clinical workflow represents one of the main challenges of the coming years [20,224].

One aspect of integration is defining the clinical contexts in which liquid biopsies add value. In advanced breast cancer, plasma-based genotyping for actionable mutations has already made inroads (for example, detecting PIK3CA mutations in ctDNA to guide targeted therapy when tumor tissue is unavailable). However, to systematically incorporate multi-omics, clinicians need clear guidelines on when to order these tests and how to act on the results [124]. Clinical trials are beginning to supply this evidence. For instance, in metastatic hormone receptor-positive breast cancer, the phase III PADA-1 trial demonstrated that acting on a dynamic liquid biopsy result can improve outcomes: patients who had an ESR1 mutation detected in blood and were switched early to a different therapy had significantly prolonged progression-free survival [44].

Another facet of integration is logistical and informatic. Hospital systems will need to accommodate frequent blood sampling, rapid processing, and analysis of multi-omic data, all within the pace of clinical practice [197,198]. This may require on-site facilities or reliable send-out workflows, as well as training for staff in handling specimens properly (tying back to standardization). The volume of data from multi-omic also demands robust bioinformatics pipelines that can distill actionable information for the oncologist. To integrate into decision-making, the results should be reported in a clear, clinician-friendly manner rather than overwhelming raw data. The need for user-friendly interpretation is driving development of clinical decision support tools that take multi-omic input and provide treatment recommendations or risk assessments [20,229,231].

Moreover, integrating liquid biopsy requires demonstrating cost-effectiveness and workflow efficiency. Busy oncology clinics cannot adopt a technology that slows down care or leads to indeterminate results. Thus, trials and implementation studies are also examining turnaround times and how often liquid biopsy results change management [25,43,232]. Early evidence is promising that liquid biopsies can be obtained and analyzed quickly, often faster than invasive tissue biopsies or radiologic assessments, thereby enabling more rapid treatment adjustments [229]. In addition, liquid biopsies can reduce the need for some invasive procedures; for example, rather than biopsy a difficult metastatic lesion, a blood test might suffice to genotype the tumor. Over time, this could streamline care and reduce patient burden. However, care must be taken to integrate these tests equitably [18,233,234].

### 6.3. Personalized Screening and Dynamic Monitoring Models

Perhaps the most exciting future application of multi-omic liquid biopsy in breast cancer is the move toward personalized screening and dynamic disease monitoring. Traditional breast cancer screening (e.g., annual or biennial mammography for women over a certain age) is largely one-size-fits-all. Likewise, follow-up after treatment is often on a fixed schedule of imaging and clinic visits. Multi-omic biomarkers open the door to more individualized approaches [235].

On the screening front, researchers are exploring blood-based tests that could detect breast cancer at its earliest stages, potentially complementing or even augmenting imaging [236]. One concept garnering attention is multi-cancer early detection (MCED) assays, which look for molecular signals of cancer in asymptomatic individuals. These tests analyze combinations of markers—such as ctDNA mutations, DNA methylation patterns, and protein biomarkers—to signal the presence of a cancer and even hint at its tissue of origin [94,226].

The Galleri test, developed by Grail, represents one of the most advanced examples of multi-cancer early detection (MCED) through liquid biopsy. The Galleri test (Grail) is a multi-cancer early detection assay that analyzes cfDNA methylation patterns to identify over 50 cancer types and predict their tissue of origin using machine learning [237]. In the PATHFINDER trial, it detected cancer signals in one-third of those who tested positive, while about two-thirds were false positives [238]. While promising as a complement to conventional screening, experts caution that its clinical benefit remains unproven, as no trial has yet shown a reduction in cancer mortality. Concerns include false positives, overdiagnosis, and limited sensitivity for early-stage cancers, particularly breast and prostate. Additional issues involve high costs, lack of insurance coverage, and potential inequities in access. Researchers stress the need for clear diagnostic follow-up pathways, further validation through randomized studies, and regulatory oversight before integrating MCED tests like Galleri into standard screening programs [239,240].

In breast cancer specifically, ongoing research is evaluating whether adding a blood-based multi-omic test could improve detection of aggressive cancers between regular mammograms or in younger women not covered by standard screening [241]. Such an approach could be personalized by tailoring screening frequency to individual risk. A woman with high genetic risk or subtle positive biomarker findings might be monitored more frequently or with adjunct tests, whereas someone with consistently negative multi-omic profiles might safely extend the interval between screenings. However, these strategies remain investigational [241]. It will be crucial to prove that blood-based screening can reduce mortality and avoid excessive false positives. No MCED test is yet approved for general population screening, underscoring the need for large prospective trials. Nonetheless, the concept of personalized screening, using multi-omic data (genomic, epigenomic, protein markers) alongside traditional risk factors to guide who to screen, for what, and how often, is a compelling future direction in breast cancer prevention [242,243].

In parallel, dynamic monitoring of breast cancer patients using liquid biopsy promises to revolutionize how we surveil disease and make treatment decisions. Unlike static snapshots (e.g., a scan every 6 months), dynamic monitoring would entail regular blood tests to continuously track tumor-derived biomarkers, enabling an up-to-the-moment picture of tumor status. This approach is particularly relevant for minimal residual disease (MRD) detection and early relapse prediction after curative treatment. Several studies have shown that ctDNA can serve as a very sensitive marker of residual disease well before any clinical or radiographic signs of recurrence [244,245]. For instance, recent clinical research in early breast cancer demonstrated that a personalized, plasma-only ctDNA assay—incorporating both tumor-specific mutations and methylation profiles—could detect recurrence in the majority of patients months in advance [244]. In a 2025 pilot study by Janni et al., ctDNA was detected at or before recurrence in 11 of 14 patients (79%), demonstrating a sensitivity of 85% for samples collected within two years of relapse. Among patients with detectable ctDNA prior to recurrence, the lead time ranged from 3.4 to 18.5 months, indicating that ctDNA can reveal recurrence months before imaging or symptoms. Importantly, no ctDNA was detected in patients without recurrence (n = 13), supporting the test’s high specificity [244]. This proof-of-concept illustrates that dynamic blood monitoring can identify high-risk patients long before conventional follow-up would catch the relapse. Armed with such early warning, clinicians could intensify surveillance or initiate treatment (e.g., start systemic therapy for impending relapse) sooner, potentially improving outcomes. Large trials (BESPOKE CRC and c-TRAK studies) are further evaluating whether ctDNA-guided interventions can actually prevent or delay metastatic recurrence in breast cancer [246,247].

## 7. Conclusions

Liquid biopsy and multi-omic biomarker technologies are reshaping breast cancer precision medicine by enabling dynamic, minimally invasive tumor assessment. Through the analysis of circulating tumor DNA, circulating tumor cells, and extracellular vesicles, clinicians can monitor tumor evolution, therapeutic response, and relapse risk in real time; capabilities that traditional tissue biopsy cannot offer. When combined with artificial intelligence and machine learning, multi-omic data provide a comprehensive molecular profile that supports earlier intervention and more personalized treatment strategies.

Despite these advances, several limitations continue to hinder widespread clinical adoption. Pre-analytical variability in sample collection, processing, and assay standardization remains a major challenge, as inconsistencies can compromise data accuracy. The high cost of multi-omic testing, limited reimbursement frameworks, and unequal access across health systems further restrict integration into routine care. Additionally, while many studies demonstrate impressive analytical performance, definitive evidence that liquid biopsy–based monitoring improves survival or reduces mortality is still limited. Ethical and logistical considerations, such as patient consent, data privacy, and the management of incidental findings, must also be addressed as molecular diagnostics become more prevalent.

The future of breast cancer management will likely involve hybrid models that combine conventional imaging with molecular surveillance. Liquid biopsy will serve as a complement to, rather than a replacement for current diagnostic standards, offering earlier insight into treatment resistance and disease recurrence. As sequencing costs decline and analytic platforms mature, multi-omic integration will extend beyond genomics to include transcriptomic, proteomic, and metabolomic signatures, allowing for more precise risk stratification and adaptive therapy design. In younger women and those with dense breast tissue (populations less effectively screened by mammography), circulating biomarkers may also provide valuable adjunctive tools for early detection.

Realizing the full clinical potential of liquid biopsy requires coordinated progress across disciplines. Hospitals must develop infrastructure to support frequent blood sampling, rapid processing, and high-throughput data analysis, while ensuring that results are translated into clear, actionable clinical reports. The development of user-friendly decision-support tools will be critical to help physicians interpret complex multi-omic outputs and integrate them into treatment planning. Equally important is the creation of large, standardized, and interoperable datasets that link molecular patterns with clinical outcomes, enabling validation across diverse patient populations.

Ultimately, liquid biopsy and multi-omic approaches represent a pivotal step toward real-time, data-driven oncology. They offer the promise of earlier detection, more precise treatment adjustments, and improved long-term outcomes. Achieving this vision will depend on sustained collaboration among clinicians, researchers, data scientists, and policy makers to ensure that technological progress translates into tangible patient benefit, delivering a future where breast cancer care is not only personalized but truly predictive and adaptive.

## Figures and Tables

**Table 1:** Commercially Available Liquid Biopsy Assays: Analytical Capabilities, Sensitivity, and Practical Considerations.

Commercial Assay	Type of Analysis	Sensitivity	Detects CNV/CNA	Detects RNA	Detects Fusions	Advantages	Disadvantages
Guardant360 CDx	NGS ctDNA, 74 genes	LLOD ~0.1% VAF	Yes	No	Yes	FDA-approved, broad panel	Does not detect RNA
FoundationOne Liquid CDx	NGS ctDNA, 324 genes	LLOD ~0.4% VAF	Yes	No	Yes	Extensive variant catalog	Lower sensitivity than Signatera
Signatera (Natera)	Personalized MRD	0.01% VAF	Limited	No	No	Highest sensitivity for MRD	Requires tumor tissue
TruSight Oncology 500 ctDNA	Broad ctDNA NGS panel	~0.5% VAF	Yes	No	Yes	Wide genomic coverage	Not clinically approved
Oncomine cfDNA/cfRNA	cfDNA + cfRNA	0.1–0.5% VAF	Yes	Yes	Yes	True RNA detection, excellent for fusions	Limited regional availability
cobas EGFR Mutation Test v2	Digital PCR ctDNA	~0.1% VAF	No	No	No	Fast and inexpensive	EGFR only
RARE-seq (cfRNA)	High-fidelity cfRNA	High (superior for fusions)	No	Yes	Yes	High sensitivity for cryptic fusions	Pre-commercial, limited availability

**Table 2:** Overview of circulating biomarkers, biological sources, clinical relevance, and validation stage.

	Molecular Composition and Origin	Clinical Relevance	Validation Stage
ctDNA/cfDNA	Tumor-derived DNA fragments released via apoptosis, necrosis, or secretion; carry PIK3CA, ESR1, TP53, HER2 mutations and methylation patterns.	Enables MRD detection, early relapse prediction (3–6 mo before imaging), and tracking of clonal evolution or therapy resistance.	ddPCR, NGS (CAPP-seq, SafeSeqS); sensitivity ≈ 93%, specificity ≈ 100%; phase II–III validation, Signatera™ (Natera, Inc., Austin, TX, USA) FDA-cleared [39,84].
Circulating Tumor Cells (CTCs)	Viable EpCAM+/CK+/CD45− cells shed into circulation; display epithelial–mesenchymal plasticity.	≥5 CTCs/7.5 mL predicts poor OS/PFS; allow real-time receptor profiling and detection of HER2 discordance.	CellSearch^®^ (Menarini Silicon Biosystems, Inc., Huntington Valley, PA, USA) (FDA-cleared); emerging microfluidic and label-free technologies; phase III validation [39,84].
Non-coding RNAs and Exosomes	40–150 nm vesicles enriched in miR-21, miR-155, lncRNA HAGLROS/HOTAIR; mediate EMT, immune modulation.	Biomarkers of drug resistance and tumor–immune crosstalk; HAGLROS promotes EMT and M2 polarization (miR-135b-3p/COL10A1).	Isolation via ultracentrifugation/affinity; analysis by RT-qPCR, RNA-seq; translational validation ongoing [69].
Proteomic and Metabolomic Signatures	Circulating proteins/metabolites (e.g., TALDO1, glycolytic/lipid intermediates) reflecting metabolic rewiring.	Distinguish localized vs. metastatic disease; TALDO1 acts as diagnostic marker and drug target.	LC-MS/MS, DIA-MS, NMR; sens. > 85%, spec. ≈ 90%; preclinical–clinical validation [130,131].

Circulating tumor DNA (ctDNA) and cell-free DNA (cfDNA) provide insight into clonal evolution and therapy resistance, enabling detection of minimal residual disease and early relapse through highly sensitive next-generation sequencing and digital PCR assays. Circulating tumor cells (CTCs) allow real-time assessment of receptor discordance and disease progression, while exosomal non-coding RNAs such as *miR-21*, *miR-155*, *HAGLROS*, and *HOTAIR* serve as biomarkers of immune modulation and drug resistance. Finally, proteomic and metabolomic signatures reflect metabolic reprogramming and can distinguish localized from metastatic disease. Together, these biomarkers represent complementary, minimally invasive tools at varying stages of clinical validation that collectively enhance precision monitoring in breast cancer [39,69,84,130,131].

## Data Availability

No new data were created or analyzed in this study.
